# Analysis of the Correlation between the Radioactive Iodine Activity and Neutrophil-to-Lymphocyte Ratio in Patients with Differentiated Thyroid Cancer

**DOI:** 10.3390/cancers14081899

**Published:** 2022-04-09

**Authors:** Adina Elena Stanciu, Andreea Verzia, Marcel Marian Stanciu, Anca Zamfirescu, Dan Cristian Gheorghe

**Affiliations:** 1 Department of Carcinogenesis and Molecular Biology, Institute of Oncology Bucharest, 022328 Bucharest, Romania; 2 Cernavoda Nuclear Power Plant Division, Nuclearelectrica, 905200 Cernavoda, Romania; andreeaverzia@yahoo.com; 3 Electrical Engineering Faculty, University Politehnica of Bucharest, 060042 Bucharest, Romania; marcel.stanciu@upb.ro; 4 Department of Radionuclide Therapy, Institute of Oncology Bucharest, 022328 Bucharest, Romania; ancacvi@gmail.com or; 5 ENT Department, University of Medicine and Pharmacy Carol Davila Bucharest, 050474 Bucharest, Romania; gheorghe.dancristian@gmail.com

**Keywords:** differentiated thyroid cancer, radioactive iodine, blood activity, neutrophil-to-lymphocyte ratio

## Abstract

**Simple Summary:**

A higher tumor burden in patients with differentiated thyroid cancer undergoing radioactive iodine (^131^I) therapy may release into the bloodstream large amounts of ^131^I with a long residence time. We hypothesized that after the ^131^I intake, the blood concentration of ^131^I shows a course in several phases. To our knowledge, this research is the first of its kind. The results of the current study demonstrated a ^131^I blood concentration biphasic course. The time points to be considered are 46 and 69 h. ^131^I uptake in the residual thyroid tissue peaked after 46 h. The positive correlation between the neutrophil-to-lymphocyte ratio and administered ^131^I activity or the blood activity shows that the time interval between 46 and 69 h should be associated with the release of hematological inflammatory mediators, such as neutrophils and lymphocytes, to eradicate tumor cells in response to ^131^I therapy.

**Abstract:**

Publications investigating the effect of radioactive iodine (^131^I) therapy on the circulating peripheral blood cells in patients with differentiated thyroid cancer (DTC) are limited to blood samples collected more than 92 h after ^131^I. Studies conducted on blood samples collected up to 92 h are rare due to the radioactive contamination risk. This research aimed to assess the relationship between the prescribed ^131^I activity, human whole blood activity, and peripheral blood cells at many time points (6, 22, 46, 69, and 92 h after ^131^I). The study enrolled 50 female patients with DTC who received a ^131^I median activity of 90.54 mCi (3.35 GBq). The neutrophil-to-lymphocyte ratio (NLR) was measured as an inflammatory marker. ^131^I uptake in the residual thyroid tissue peaked after 46 h. Blood activity decreased in the first 46 h and increased 69 h after the ^131^I intake. Blood activity was associated with the absolute lymphocyte count and the NLR at 69 h (r = −0.49 and r = 0.52, *p* < 0.001). Our results demonstrate that the time interval between 46 and 69 h should be associated with the release of hematological inflammatory mediators, such as neutrophils and lymphocytes, to eradicate tumor cells in response to ^131^I therapy.

## 1. Introduction

Differentiated thyroid cancer (DTC), the most common endocrine malignancy, accounts for more than 90% of all thyroid cancers. Incidences of DTC have been steadily increasing over the last decades [1]. According to a joint statement from the European Thyroid Association (ETA), American Thyroid Association (ATA), European Association of Nuclear Medicine (EANM), and Society of Nuclear Medicine and Molecular Imaging (SNMMI), targeted therapy with radioactive iodine (^131^I) is the standard procedure for (i) remnant ablation (the destruction of remaining, presumably benign, residual thyroid tissue); (ii) adjuvant treatment (the destruction of suspected, but not identified, post-operatively remaining iodine-avid primary tumor tissue and metastatic foci); or (iii) treatment of known disease (the destruction of identified residual or recurrent disease) [2,3].

The radionuclide ^131^I simultaneously emits two types of radiation (beta minus radiation for intracellular therapy and gamma radiation for diagnosing the presence or absence of remnant thyroid tissue) [4]. The physical half-life of ^131^I is about 8.05 days. Regarding the ^131^I biological half-life, there are significant differences between healthy people, patients with hyperthyroidism or hypothyroidism, and patients with DTC who underwent total thyroidectomy [5,6]. A five-compartmental model (stomach, body fluid, thyroid, whole-body, and excretion) was established to simulate the metabolic mechanism of ^131^I, according to Eckeman’s original suggestion [5] and ICRP-56 report recommendations [6]. The average biological half-life values for the stomach, body fluid, thyroid, and whole-body proposed by Huang et al. [7] in DTC patients who underwent post-thyroid cancer remnant ablation were 0.54 ± 0.32 h, 12.6 ± 1.8 h, 42.8 ± 5.1 h, and 12.6 ± 1.8 h, respectively.

As all DTC patients have some remaining thyroid tissue after thyroidectomy, strong uptake of ^131^I into the thyroid bed is a prerequisite for achieving the three ^131^I therapy goals. A high serum level (over 30 IU/mL) of the thyroid-stimulating hormone (TSH) is also required [2,3]. This can be achieved either by thyroid hormone withdrawal or by injection of recombinant human TSH (rhTSH).

Blood cell interactions are essential in the pathophysiology of inflammation and immune responses in DTC patients [4]. These interactions have many facets, and it is often difficult to distinguish the specific roles of each cell type in response to high doses of ^131^I. It is also challenging to say when these interactions are triggered. The most common circulating white blood cells are neutrophils, and the most radiosensitive human blood cell populations are lymphocytes. Neutrophils and lymphocytes exhibit antitumor effects [4,5,6,7,8]. The neutrophil-to-lymphocyte ratio (NLR) in peripheral blood reflects the imbalance between immune surveillance and tumor progression before and after ^131^I therapy [9,10,11]. However, the relationship between the therapeutic dose of ^131^I and the NLR is unknown.

Publications investigating the effect of systemic internal irradiation with ^131^I on the circulating peripheral blood cells in patients with DTC are limited to blood samples collected at intervals of more than 92 h after the administration of the therapeutic dose [12,13]. Studies performed on blood samples collected up to 92 h are rare due to the risk of radioactive contamination.

This study aimed to assess the relationship between the prescribed ^131^I activity, human whole blood activity, and peripheral blood cells at many time points after internal radiotherapy (5-time points as follows: 6, 22, 46, 69, and 92 h).

## 2. Materials and Methods

### 2.1. Patients and Study Protocol

Fifty female patients with DTC (mean age 58.98 ± 10.85) were admitted for ^131^I therapy in the Department of Radionuclide Therapy of the Institute of Oncology Bucharest. The patients enrolled in the study were treated with a total thyroidectomy, followed by the targeted therapy with ^131^I sodium iodide ThyroTop. ^131^I sodium iodide ThyroTop is a radiopharmaceutical purchased from the Institute of Isotopes Co. Ltd. (IZOTOP), Budapest, Hungary. Indication for ^131^I therapy in this cohort of patients was based on the recommendation from guidelines developed by the ETA and ATA [14,15], and the joint statement from the ETA, ATA, EANM, and SNMMI [3] in compliance with safety measures [16]. Levothyroxine (L-T4) was discontinued 4–6 weeks and triiodothyronine (L-T3) two weeks prior to therapy with ^131^I sodium iodide ThyroTop. A diet low in iodine for two weeks was recommended for all patients before ^131^I administration. Patients were advised to drink sufficient fluids (3–4 L/day) after receiving the therapeutic dose of ^131^I sodium iodide ThyroTop to enable frequent urination for faster elimination of the ^131^I from the body.

The exclusion criteria were as follows: (i) age under 18 years; (ii) renal impairment (kidney function is involved in ^131^I elimination) [17]; (iii) gastrointestinal disease (stool frequency); (iv) ongoing treatment with diuretics (urine frequency); (v) receiving external beam radiotherapy and/or chemotherapy at any time; (vi) concurrent or pretreatment hematologic malignancies; (vii) ongoing treatment with steroidal anti-inflammatory drugs (known to affect the absolute lymphocyte count) [18]. Only women were included in the study to avoid intersex variations. For each enrolled patient, a detailed medical and drug history was obtained.

The study was conducted following the principles outlined in the Declaration of Helsinki and was approved by the Institute of Oncology Bucharest Medical Ethics Committee (approval No. 15140/10.09.2019). All the patients were informed about the study goals and the medical procedures for blood sampling and whole-body dose rate measurement. Signed informed consents were obtained from all subjects at their admission.

### 2.2. Radioactive Blood Sampling

Peripheral blood specimens were collected by venipuncture into BD Vacutainer EDTA tubes at different time intervals (6, 22, 46, 69, and 92 h) after the ^131^I therapeutic dose administration. The volume of blood was 2 mL. It should be noted that all blood samples collected after the ^131^I intake were radioactive. Therefore, they were labeled with a sticker displaying the international symbol of radioactivity. A separate procedure for the radioactive biological samples of collection, transport, processing, and removal was prepared. This procedure followed the rules in force developed by the guidelines of the Dosimetry Committee of the European Nuclear Medicine Association (EANM) [19] and ISO 15189: 2013/ISO 15190: 2005 (requirements for quality and competence in medical laboratories/requirements for safety in medical laboratories).

### 2.3. Blood Volume and Body Mass Index Calculation

The patients’ blood volume and body mass index (BMI) were calculated according to their height (*H*) and weight (*W*). The Nadler equation for women: ${Blood}_{Volume}=\left(0.3561xH^{3}\right)+\left(0.03308xW\right)+0.1833$ was used to calculate the blood volume. The BMI value was obtained with the formula BMI = *W*/*H*^2^. The healthy weight range considered for BMI was 18.5 to 24.9 kg/m^2^. A BMI of 25 to 29.9 kg/m^2^ is considered overweight, and greater than 30 kg/m^2^ is considered obese (obesity class I: BMI = 30 to 34.9 kg/m^2^; obesity class II: BMI = 35 to 39.9 kg/m^2^; and obesity class III: BMI greater than or equal to 40 kg/m^2^) [20].

### 2.4. Whole Blood Activity Measurement

The activity of each blood sample (2 mL) was measured with a dose calibrator (CURIEMENTOR^R^ 4 Isotope Calibrator, PTW Freiburg, Germany). The background count was measured before measuring the activity of each blood sample. The measurement accuracy was approximately 5%, ranging from 0.01 µCi to 2486 mCi (or from 0.001 mBq to 92 GBq). Further, the activity measured with the CURIEMENTORR 4 Isotope Calibrator was divided by 2 to obtain blood sample activity/1 mL. Next, each patient’s whole blood activity was calculated by multiplying the blood sample activity/1 mL by patient blood volume.

### 2.5. Whole-Body Dose Rate Measurement

**Figure 1:**
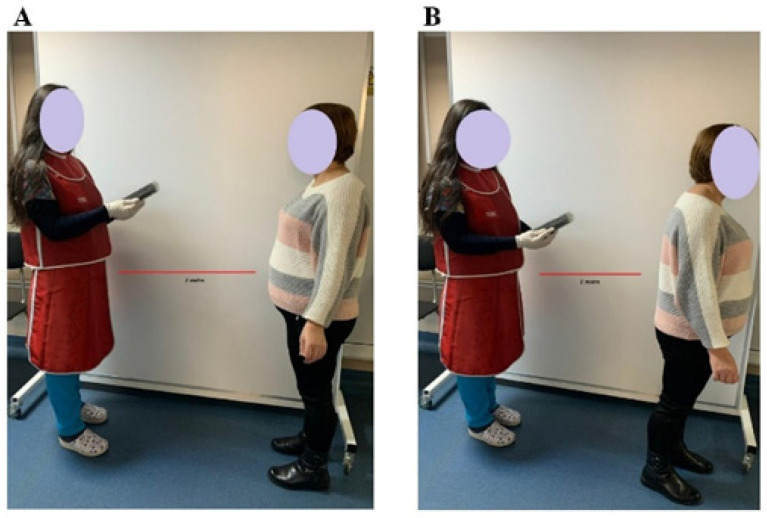
Radioactivity measurements performed at a distance of 1 m from the patient’s abdomen (**A**) frontal position; (**B**) posterior position.

Each patient enrolled in the study underwent whole-body measurements at 6, 22, 46, 69, and 92 h after the ^131^I intake, with a full bladder, and after that, with an empty bladder, using a high sensitivity digital radiation meter and dosimeter (Thermo FH 40 G-L10 Survey Meter, PTW Freiburg, Germany). The first data after 6 h corresponded to the effective administered activity of ^131^I. Three measurements were performed at a distance of 1 m for the neck (thyroid), abdomen (stomach), and legs, both frontally and posteriorly, as shown in Figure 1 with the abdominal area. Then, the mean was calculated after background subtracting for each set of values. Measurements are given in terms of ambient dose equivalent rate (µSv/h).

### 2.6. Biomarker Measurement

Blood-cell analysis (blood-count parameters: neutrophils and lymphocytes) was performed on an ADVIA 2120i automatic Hematology System with auto slide (Siemens). The NLR was calculated by dividing the absolute neutrophil count by the absolute lymphocyte count. The serum TSH analysis was performed on integrated clinical chemistry and immunoassay Alinity ci-series (Abbott) from blood specimens collected by venipuncture into Vacutainer (Becton Dickinson, Rutherford, NJ, USA) serum separation tubes (6 mL) before the ^131^I intake.

### 2.7. Statistics

Microsoft Office Excel 2007 SP2 (including Data Analysis) was used for patients’ data processing. Statistical analysis was conducted with Statistica software (version 8.0; StatSoft, Inc., Tulsa, OK, USA). Continuous variables were expressed as median values with an interquartile range (IQR: 25–75%). Shapiro–Wilk and Kolmogorov–Smirnov tests were used to verify the data obtained after preliminary analysis and check the consistency of the group. Pearson’s correlation coefficient (r) was used to assess the relationships between the measured variables. Significance was set at a *p*-value < 0.05.

## 3. Results

### 3.1. Characteristics of the Study Population

Clinical and biochemical data are summarized in Table 1. The median BMI of 30.21 kg/m^2^ showed that female patients with DTC suffer from class I obesity (the BMI value was 30–34.9 kg/m^2^). According to their height and weight, the median volume of blood/patient was estimated at 4454 mL. The median TSH level of 81.3 mIU/L confirmed the short hypothyroid state, specific to thyroid hormone withdrawal. The patients enrolled in the study were treated with a median dose of ^131^I sodium iodide ThyroTop equal to 90.54 mCi (IQR: 63.20–132.97 mCi) [3.35 GBq (IQR: 2.34–4.92 GBq)].

### 3.2. Variation over Time of the Blood Activity

**Figure 2:**
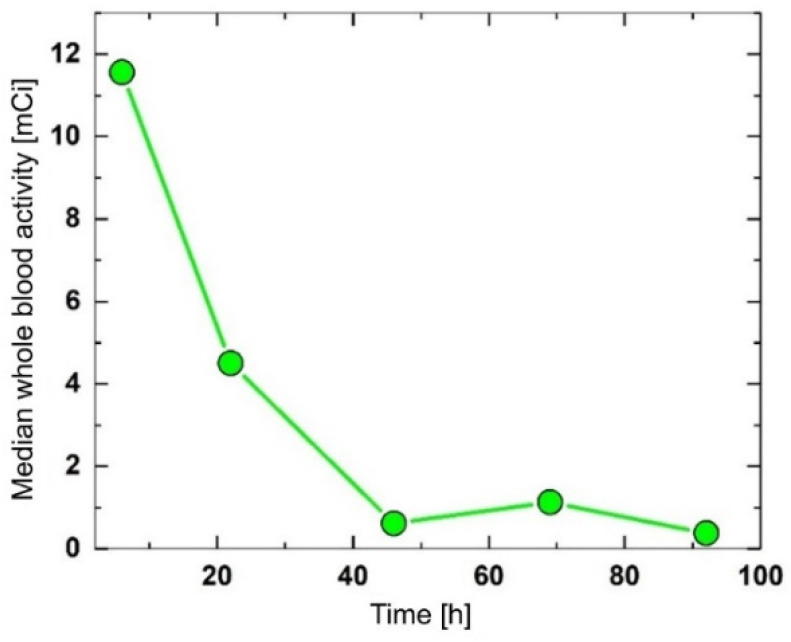
Blood activity curve as a function of time.

The blood sampling activity obtained at 6, 22, 46, 69, and 92 h after the ^131^I administration is shown in Figure 2. The time-activity curve shows a decreasing trend with a peak at 69 h. Whole blood activity, and implicitly, blood inorganic ^131^I, rapidly decreased in the first 46 h, which was most likely due to the fast clearance by the kidneys. The 69-h peak may be due to a protein-bound ^131^I released by the remnant thyroid tissue.

### 3.3. Variation over Time of the Dose Rate

**Figure 3:**
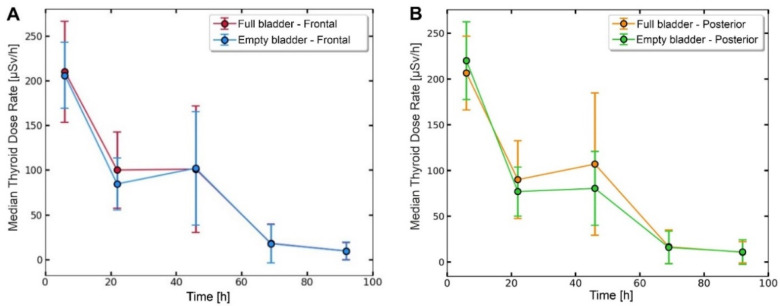
Variation over time of the dose rate measured at 1 m distance from the neck area (**A**) frontal position; (**B**) posterior position.

The dose rate measured at 1 m from the neck area (Figure 3A—frontal position and Figure 3B—posterior position) decreased over time. A slight change could be observed after urination (empty bladder). As shown in Figure 3, the uptake of ^131^I into the thyroid bed peaked at 46 h after the oral administration. According to the five-compartment model (stomach, body fluid, thyroid, whole-body, and excretion) designed to explain the metabolic mechanism of ^131^I [5,6,7], after oral administration, ^131^I sodium iodide was rapidly absorbed by the gastrointestinal tract, then passed into the extracellular fluid, and after that, into the extrathyroidal compartment where it organically bound to the remnant thyroid tissue.

### 3.4. Correlations

**Figure 4:**
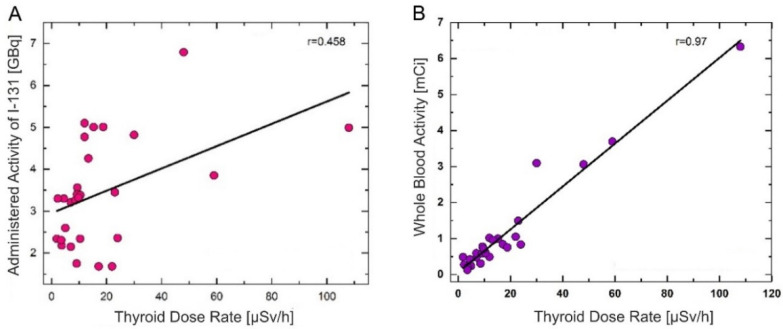
Correlation measured at 46 h between the dose rate at 1 m distance from the neck area and ^131^I activity (**A**); correlation measured at 69 h between the dose rate at 1 m distance from the neck area and the whole blood activity (**B**).

As shown in Figure 4A (r = 0.458, *p* < 0.001), the correlation between the administered ^131^I activity and the dose rate at a 1 m distance from the thyroid was statistically significant only at 46 h after the ^131^I intake, most likely because the sodium iodide ^131^I uptake in the remnant thyroid tissue reaches a maximum after 46 h. Regarding the whole blood activity, the strongest correlation between the dose rate at a 1 m distance from the thyroid and the blood activity was measured only at 69 h post ^131^I intake, when ^131^I was released in the blood by the residual thyroid tissue (Figure 4B: r = 0.97, *p* < 0.001).

**Figure 5:**
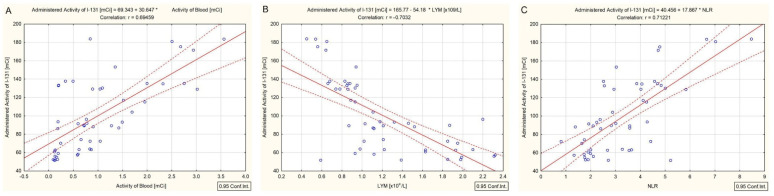
Correlation measured at 69 h post-administration between the ^131^I activity and whole blood activity (**A**), absolute lymphocytes count (**B**) and NLR (**C**).

Lymphocytes and neutrophils analysis revealed that the best association between these blood-count parameters and the ^131^I activity or the whole blood activity was calculated at 69 h after the therapeutic ^131^I administration. As a confirmation, the ^131^I activity was positively correlated with the whole blood activity (r = 0.69, *p* < 0.001) and the NLR (r = 0.71, *p* < 0.001) (Figure 5A,C), and negatively with the absolute lymphocytes count (r = −0.70, *p* < 0.001) (Figure 5B). No correlation between the administered ^131^I activity and absolute neutrophils count was noticed.

**Figure 6:**
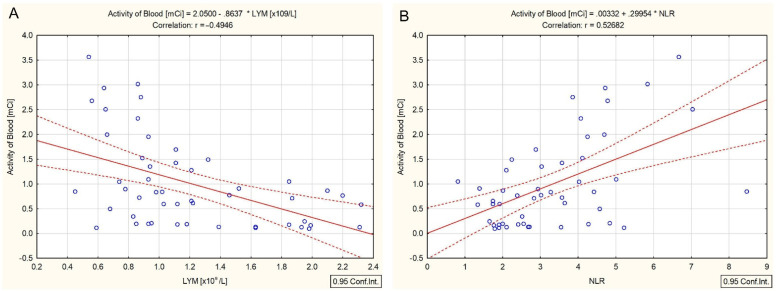
Correlation measured at 69 h post-administration between the whole blood activity and absolute lymphocytes count (**A**) and NLR (**B**).

Moreover, there was a negative correlation between the whole blood activity and the absolute lymphocytes count (r = −0.49, *p* < 0.001) (Figure 6A), and a positive one with the NLR (r = 0.52, *p* < 0.001) (Figure 6B).

Basically, the time interval between 46 and 69 h could be associated with the release of hematological inflammatory mediators, such as neutrophils and lymphocytes, to eradicate tumor cells in response to ^131^I therapy. This time interval may be of interest in treating patients who do not respond very well to ^131^I.

## 4. Discussion

To our knowledge, this is the first study to report the relationship between the prescribed ^131^I activity, human whole blood activity, and the NLR at many time points after internal radiotherapy (5-time points). Specifically, the main findings are: (i) sodium iodide ^131^I uptake in the residual thyroid tissue reaches a maximum after 46 h; (ii) the whole blood activity decreases in the first 46 h and increases at 69 h after the ^131^I intake; (iii) the whole blood activity is associated with the dose rate measured at a 1 m distance from the thyroid area and with the prescribed ^131^I activity at 69 h post-administration; (iv) the whole blood activity is negatively associated with the absolute lymphocyte count and positively with the NLR at 69 h after ^131^I administration.

Some studies [12,13] have suggested that a higher tumor burden may release into the bloodstream large amounts of ^131^I with a long residence time, without explicitly demonstrating this. We hypothesized that after the ^131^I intake, the blood concentration of ^131^I shows a course in several phases.

The dose rate emitted at a distance of 1 m by a patient treated with radiopharmaceuticals is a marked parameter in radiation protection calculations. The dose rate was measured in three areas of interest (neck, abdomen, legs) from the anterior and posterior positions, as shown in Figure 1 for the abdominal area. The measurements considered the urinary bladder (full or empty) because a percentage between 50 and 75% of the administered radioactivity was excreted in urine (Figure 3). After oral administration, sodium iodide ^131^I is quickly absorbed by the upper gastrointestinal tract (90% in 1 h) [21]. The sodium iodide ^131^I uptake is affected by gastric emptying. From the gastrointestinal tract, sodium iodide ^131^I passes into the extracellular fluids being distributed in the extrathyroidal compartment, where it is taken up by the residual thyroid tissue that extracts approximately 20% of the iodide in one pass. Iodide is further oxidized to iodine, which organically binds remnant thyroid tissue [22,23]. Our results, presented in Figure 3, show that the sodium iodide ^131^I uptake in the residual thyroid tissue reached a maximum after 46 h. Moreover, this “maximum” expressed by the dose rate measured at 1 m from the thyroid was statistically significantly associated with the administered ^131^I activity, as shown in Figure 4A (r = 0.458, *p* < 0.001). This finding agrees well with a previous study conducted by Nichols et al. [24] on a group of 65 patients with thyroid cancer. The 46-h interval can be considered the first phase of the ^131^I blood concentration course.

Moreover, the sustained presence of a high serum TSH level (median concentration: 81.3 mIU/L), achieved by the L-T4 treatment discontinuation for 4–6 weeks before ^131^I therapy (short hypothyroid state), is responsible for increased absorption in the remnant thyroid cells. The long residence time in the thyroid tissue led to a higher dose absorbed into the blood. Our results revealed a rapid decrease in the whole blood activity in the first 46 h and a slight increase at 69 h after the ^131^I intake, as shown in Figure 2. In the first 24–46 h, a rapid decrease in inorganic ^131^I in the blood, demonstrated by a marked decrease in the whole blood activity, was most likely due to fast clearance by the kidneys. In the next 23 h, a protein-bound ^131^I peak, expressed as an increased whole blood activity, was due to be released by the residual thyroid tissue. Moreover, the correlation, measured at 69 h post-administration of the ^131^I activity between the dose rate at a 1 m distance from the neck area and the whole blood activity (r = 0.97, *p* < 0.001), comes to prove the existence of a second phase of the ^131^I blood concentration course. Increased whole blood activity and the significant correlation with the dose rate at a 1 m distance from the neck area demonstrate that at 69 h, ^131^I was released in the blood by the residual thyroid tissue. In addition, at 69 h the whole blood activity was strongly associated with the prescribed ^131^I activity (r = 0.69, *p* < 0.001), as shown in Figure 5A. Our results are in accordance with those obtained by Piruzan et al. [25], who noted that the absorbed dose to the blood was correlated with the blood sampling activity after at least 48 h post-administration (r = 0.62 and *p* < 0.001).

The number of publications investigating the effect of systemic internal irradiation with ^131^I on the circulating peripheral blood cells in patients with DTC is still limited [12,13]. Systemic internal irradiation assumes that the cells are not only irradiated by internalized and extracellular ^131^I for seconds or minutes, but are continuously irradiated over a much more extended period with a permanently changing dose rate [22]. Specifically, circulating peripheral blood cells are continually exposed to radiation doses for a long time due to the ^131^I effective half-life of about 12 h in blood plasma and about 6 days in the thyroid gland [21,26]. This exposure results in cell renewal, apoptosis, and redistributions of the hematopoietic cells [4,9,10,11,27]. Peripheral blood lymphocytes are constantly regenerating and, therefore, are the most radiosensitive human blood cell populations. The NLR reflects the immune status after radiotherapy. Several studies have reported that the NLR is an independent prognostic factor in DTC patients [9,10,11]. The positive correlation between the prescribed ^131^I activity and the NLR (r = 0.71, *p* < 0.001), and between the whole blood activity and the NLR (r = 0.52, *p* < 0.001), shows that the time interval between 46 and 69 h should be associated with the release of hematological inflammatory mediators such as neutrophils and lymphocytes to eradicate tumor cells in response to ^131^I therapy. The absolute number of lymphocytes negatively correlated with the activity of ^131^I (r = −0.70, *p* < 0.001) confirms, once again, the above.

Only women were included in the study due to sex-related differences that might appear in the interpretation of data and implicitly in the clinical outcome. Most likely, sex hormones are the main actors of the sex differences in DTC, and among them, estrogen stands out. Sex hormones can modulate the interaction between genes and the immune responses to ^131^I therapy. Progesterone has been shown to have extensive anti-inflammatory effects [28]. Its receptors are present on many different immune cells, including natural killer cells, macrophages, dendritic cells, and T cells. Because estradiol receptors are expressed in lymphoid tissue, including lymphocytes, macrophages, and dendritic cells, their presence may improve cellular and humoral mediated immune responses to ^131^I. Unfortunately, androgens (dihydrotestosterone and testosterone) present in high concentrations in men suppress the activity of immune cells, leading to low immune responses [29]. Estrogen levels are closely related to the NLR as a marker of inflammation [30]. Hormonal differences between men and women may influence the clinical outcome of ^131^I therapy, with the innate and adaptive immune responses being stronger in women than in men [28].

This study had certain limitations. The most apparent weakness was related to the small number of patients enrolled. However, despite the small sample size, the population was homogenous and included only women. Moreover, with a higher number of patients, the risk of radioactive contamination would have been even greater. A strength of our study was that all patients with steroidal anti-inflammatory drugs that could influence peripheral blood counts, as well as patients treated with external radiotherapy, were excluded from the analysis.

In summary, the results of the current study demonstrated the ^131^I blood concentration biphasic course. The time points to be considered are 46 and 69 h. The positive correlation between the NLR and administered ^131^I activity or the whole blood activity shows that the time interval between 46 and 69 h should be associated with the release of hematological inflammatory mediators, such as neutrophils and lymphocytes, to eradicate tumor cells in response to ^131^I therapy. Further investigation is required to confirm these findings.

## Figures and Tables

**Table 1:** Clinical data in the study group.

Variables	DTC
	*n* = 50
Age (years) ^a^	58.98 ± 10.85
Height (m) ^b^	1.63 (1.60–1.65)
Weight (kg) ^b^	83.5 (70.0–92.0)
BMI (kg/m^2^) ^b^	30.21 (26.56–34.37)
Blood Volume (mL) ^b^	4454 (4031–4892)
TSH (mIU/L) ^b^	81.3 (63.9–99.8)
Administered Activity of ^131^I (mCi) ^b^	90.54 (63.20–132.97)

BMI, body mass index; DTC, differentiated thyroid cancer; NLR, neutrophil-to-lymphocyte ratio; ^131^I, radioiodine; TSH, thyroid stimulating hormone; ^a^ mean ± standard deviation; ^b^ data are expressed as median and interquartile ranges (25–75%).

## Data Availability

The data presented in this study are available on request from the corresponding author.
